# Early portfolio pruning: a scalable approach to hybrid portfolio selection

**DOI:** 10.1007/s10115-023-01832-7

**Published:** 2023-01-31

**Authors:** Daniele G. Gioia, Jacopo Fior, Luca Cagliero

**Affiliations:** 1 Department of Mathematical Sciences, Politecnico di Torino, Corso Duca degli Abruzzi 24, 10129 Torino, Italy; 2 Department of Control and Computer Engineering, Politecnico di Torino, Corso Duca degli Abruzzi 24, 10129 Torino, Italy

**Keywords:** Portfolio selection, Early portfolio pruning, Artificial intelligence, Decision support systems, Parallel itemset mining

## Abstract

Driving the decisions of stock market investors is among the most challenging financial research problems. Markowitz’s approach to portfolio selection models stock profitability and risk level through a mean–variance model, which involves estimating a very large number of parameters. In addition to requiring considerable computational effort, this raises serious concerns about the reliability of the model in real-world scenarios. This paper presents a hybrid approach that combines itemset extraction with portfolio selection. We propose to adapt Markowitz’s model logic to deal with sets of candidate portfolios rather than with single stocks. We overcome some of the known issues of the Markovitz model as follows: (i) *Complexity*: we reduce the model complexity, in terms of parameter estimation, by studying the interactions among stocks within a shortlist of candidate stock portfolios previously selected by an itemset mining algorithm. (ii) *Portfolio-level constraints*: we not only perform stock-level selection, but also support the enforcement of arbitrary constraints at the portfolio level, including the properties of diversification and the fundamental indicators. (iii) *Usability*: we simplify the decision-maker’s work by proposing a decision support system that enables flexible use of domain knowledge and human-in-the-loop feedback. The experimental results, achieved on the US stock market, confirm the proposed approach’s flexibility, effectiveness, and scalability.

## Introduction

Stock portfolio selection aims at allocating funds to financial equities. The pioneering work by [1] presents the popular mean–variance model to address portfolio optimization. In a nutshell, stock return and risk of investment are quantified using first- and second-order moments of per-stock historical price distributions. Although lots of efforts have been performed by researchers to solve and expand Markowitz’s model, scalability issues [2] and reliability of the estimated values still need to be faced. In fact, the information required by [1] to estimate expected value and higher-order moments super-linearly scales with the candidate stocks [3]. More specifically, the Markowitz approach concerns the estimation of covariance values and the cardinality of the model parameters is quadratic with the number of considered assets. Estimating a so large number of model parameters, beyond requiring a considerable computational effort, raises serious questions on the reliability of these values [4]. Furthermore, investors commonly need to enforce additional, more complex constraints, e.g., incorporating transaction costs and sector-based stock diversification strategies [5]. Many of them require the adoption of heuristic methods as the new problem becomes NP-hard. For this reason, the most common portfolio selection approaches apply heuristic methods to shortlist the most relevant stocks [6–8].

The recent advances in data mining and machine learning techniques have fostered the development of *hybrid solutions* to portfolio optimization. They consist of a two-step process, where a subset of the most relevant stocks is selected first based on data-driven models and then an optimization step is applied on top of the shortlisted stocks [9]. The techniques used for stock selection at the first stage encompass, among other, machine learning and deep learning models [10], clustering techniques [11], and swarm intelligence and other metaheuristics [12]. To the best of our knowledge, all previous hybrid methods select portfolios on top of a shortlist of individual stocks. This limits the efficiency and scalability of the optimization step. To efficiently and effectively integrate complex portfolio-level constraints deeply into the portfolio selection step, it would be desirable to early prune part of the portfolio candidates at this previous stage.

The present paper proposes a scalable hybrid method, namely *early portfolio pruning* (EPP), where a set of candidate portfolios is early generated at the first step by means of an itemset-based heuristic. Then, the selection problem is no longer solved on top of a set of *single stocks*, but rather on a *portfolio shortlist*. In other words, the main analytical complexity is moved up to the itemset-based heuristic phase and accomplished by means of ad hoc, scalable algorithms. The name assigned to the presented method (EPP) emphasizes its peculiar characteristic to early discard the less interesting portfolios from the search space as soon as possible. To overcome the limitations of the Markowitz approach, we quantify the interactions among stocks only within a restricted number of portfolios previously shortlisted by an itemset mining algorithm, thus reducing the model complexity. Moreover, we provide decision-makers with a configurable system that does not forcibly depend on the first two moments, which are often contested in the financial world because of their instability and subject to great variation, thus allowing the incorporation of other metrics.

The proposed hybrid portfolio generation method allows investors to customize the selection process at their complete discretion even while coping with a large set of stocks. To this aim, we first reformulate the optimization problem by [1] to tailor it to the modified task. Then, we propose a scalable implementation of the EPP method integrating a parallel, scalable implementation of an itemset mining algorithm [13]. Finally, we integrate the presented method into a financial decision support system (DSS), which leverages fundamental data analysis to choose the portfolio according to the end-users’ preferences.

We run both performance evaluations and scalability tests on stocks belonging to the US market. The outcomes of the backtesting simulations confirm the effectiveness of EPP compared to previous approaches: EPP produces, on average, a good combination of profitable yet low volatile portfolios, also in adverse market conditions (e.g., during the COVID-19 pandemic). Furthermore, the scalable implementation allows EPP to handle stock sets not manageable by existing itemset-based heuristics, e.g., [14].

**Fig. 1:**
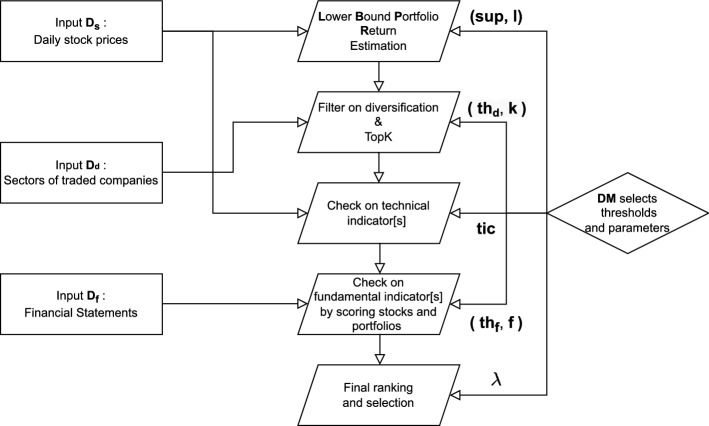
Graphical representation of the main decision support system steps

The key contributions of the paper are enumerated below:

- We present EPP, a hybrid method to select stock portfolios where candidate portfolios are early pruned by means of an itemset mining process.
- We propose an adapted version of the established mean–variance logic proposed by [1]. The proposed approach partly overcomes the inherent complexity of the traditional Markowitz model due to the excessively large number of estimated parameters as well as supports the enforcement of portfolio-level constraints on real features other than the stock prices [15].
- We present a decision support system allowing experts to monitor the portfolio selection process and leverage domain knowledge by enforcing the portfolio-level constraints (see Fig. 1).
- We adopt a parallel itemset mining implementation to perform candidate portfolio generation in a scalable way. We also empirically demonstrate the scalability of the proposed approach with the number of analyzed stocks and the size of the training time window.
- We compare EPP performance with that of both existing methods and real US funds in terms of portfolio payout (profit measure) and volatility (risk measure), showing competitive results.

The rest of the paper is organized as follows. Section 2 reviews the state of the art. Sections 3 and 4, respectively, formalize the traditional and adapted mean–variance model. Section 5 describes the architecture of the decision support system. Section 6 reports the main experimental results, whereas Sect. 7 draws conclusions and discusses future works.

## Literature review

Stock portfolio optimization aims at allocating funds to a set of selected equities [16]. The traditional mean–variance model proposed by [1] focuses on finding the best trade-off between return of investment and risk by, respectively, quantifying them as the mean and variance of the historical stock prices. Several extensions of the original model have been proposed in the literature. They propose, for example, integrating more advanced risk measurements, to handle a maximum number of selected stocks, and to incorporate transaction costs in the optimization model [15]. Another commonly used approach based on the traditional mean–variance model is the capital asset pricing model (CAPM). It is still widely used for portfolio construction, although it is often criticized for its poor empirical performance and strong simplifications, which invalidate its application use [17]. In its classic Sharpe–Lintner version, the expected return on a given asset is constructed through the risk-free interest rate plus a risk premium (market beta of the asset) multiplied by the premium per unit of beta risk.

Rather than finding the portfolio that best matches a set of restrictive conditions, portfolio optimizers can be integrated into financial decision support systems [18–20]. The aim is to allow end-users to specify their personal preferences, targets, and attitude to risk. This work presents a decision support system integrating a hybrid strategy to portfolio selection.

The research community has paid a particular attention to properly handle efficiency and estimation issues. In fact, linear and quadratic mixed-integer programming solvers may encounter issues while coping with a large number of securities. Moreover, allowing end-users to personalize the portfolio selection commonly entails enforcing ad hoc constraints, which may further increase the complexity of the optimization problem [21]. To find computationally effective solutions to NP-hard problems, the most common strategy is to adopt heuristic approaches to shortlist the candidate stocks [6–8]. Similar to [6–8], this paper addresses the selection of a subset of profitable stocks to buy, while introducing additional ad hoc constraints on the candidate portfolio.

More recently, researchers have tried to combine optimization strategies with data mining and machine learning techniques with the goal of heuristically choosing the most convenient stocks to buy. Specifically, hybrid approaches apply machine learning techniques to forecast future stock prices and then shortlist the stocks with a higher expected return to create the portfolio. For example, [22] and [3] rely on an investment decision model that predicts the direction of the stock prices first. Next, only those stocks designed to reach the expected return are considered eligible for the Markowitz optimization model. Similarly, [23] apply a genetic algorithm to select good quality assets at the first stage. This paper proposes a hybrid approach that combines established data mining techniques, i.e., itemset mining, with optimization techniques. Unlike [3, 22, 23], the proposed approach is fully unsupervised.

Other hybrid approaches rely on a two-stage process that performs stock evaluation and scoring. For example, [10] first evaluate each individual stock by performing a prediction of the stock return in the next time period. Next, they compute a scoring function that takes into account fundamental factors such as the net profit margin and the cash flow ratio. Alternative stock evaluation and scoring strategies encompass the use of clustering to find groups of similar stocks [11], genetic algorithms [24] or swarm intelligence methodologies [25–27] to deal with portfolio optimization, and itemset mining to generate candidate portfolios satisfying global constraints on the expected portfolio returns [14]. [14] perform a greedy selection of the candidate portfolios based on a set of a user-specified constraints related to portfolio size and diversification level. Unlike [10, 11] we early perform not only single stock selection but also global portfolio evaluation based on a parallel itemset mining approach. Unlike [14], on top of the itemset mining phase we shortlist the best candidate portfolio using an adapted Markowitz logic that incorporates a variety of additional constraints (including those based on fundamental analysis). Furthermore, we adopt a parallel implementation of the itemset mining process to scale toward large sets of stocks.

## Problem statement

### Notation

Hereafter, we will adopt the notation reported in Table 1.

**Table 1:** Summary of notations and their meanings

$F$	Financial statements of the candidate stocks
$S$	Set of candidate stocks
$\mathbb {P}$	Power set of $S$ that represents all the possible portfolios
$P_q$	Stock portfolio consisting of a selection of candidate stocks, indexed by $q\in \{1,\dots ,|\mathbb {P}|\}$
$H$	Historical price series of the candidate stocks within the reference time period
$w_i$	Proportion of the total amount available for investment applied to stock $s_i \in S$
$x_q$	Binary vector in $\{0,1\}^{|S|}$ with 1 when the stock $s_i$ is selected in portfolio $P_q$ and 0 otherwise
$E$[$\cdot$]	Expected value function
$E_{min}$[$\cdot$]	Lower-Bound estimate of the portfolio return (LBPR)
$R_i$	Random variable return of stock $s_i \in S$ over the holding period
$\mu _{i} = E [R_i]$	Expected return of the individual stock $s_i$ over the holding period
$\mu ^{\mathbb {P}}:\mathbb {P}\rightarrow \mathbb {N}^+$	Generalized expected return of the portfolio $P_q$ over the holding period
$\varvec{\mu }$	Vector with the expected return of all the stock in $S$
$\sigma _{ij} = Cov (s_i,s_j)$	Covariance of the returns for the pair of stocks $s_i$ and $s_j$
$\Sigma$	Covariance in matrix form for all the stock in $S$
$\Sigma ^{\mathbb {P}}:\mathbb {P}\rightarrow \mathbb {N}^+$	Generalized risk measure for the portfolio $P_q$ over the holding period
$c ^{\mathbb {P}}:\mathbb {P}\rightarrow \mathbb {N}^+$	Technical and fundamental analysis constraint function for the portfolio $P_q$ over the holding period
$\textbf{1}$, $\textbf{0}$	Vectors with all elements set to 1 and 0

### The Mean–Variance model

The original mean–variance (MV) model is among the most established stock portfolio optimization strategies [1]. The key idea is to deal with the return of a single stock as a random variable and to consider expected return and variance to model stock profitability and risk level, respectively. To quantify the return of investment and the risk level of each individual stock, the distribution descriptors are computed over the historical stock prices $H$ [28].

According to the MV model, the return of a candidate stock $s_i$ is modeled as a random variable $R_i$, with associated expected return $\mu _i = E (R_i)$. By identifying the vector of these latter values as $\varvec{\mu }$, the expected portfolio return is formulated as follows,

1 $$\begin{aligned} \begin{aligned} \varvec{\mu }^{\textsf{T}}\textbf{w} = \sum ^{|S|}_{i=1} w_i \mu _i. \end{aligned} \end{aligned}$$

Where the participation weights of the candidate stocks are stored into vector $\textbf{w} \in \mathbb {R}^{| S |}$, denoting by $w_i$, *i*=1,2,$\ldots$,$| S |$ the weight of stock $s_i \in S$ in portfolio $P$.

Beyond maximizing the expected return of the selected portfolio, the MV model incorporates portfolio diversification by estimating the return dispersion as

2 $$\begin{aligned} \textbf{w}^\textsf{T}\Sigma \textbf{w} = \sum ^{|S|}_{i=1} \sum ^{|S|}_{j=1} w_i \sigma _{ij} w_j . \end{aligned}$$

According to the MV model, the stock portfolio optimization problem can be modeled as a linear combination of the aforesaid objectives [4]

3 $$\begin{aligned} \begin{aligned} {\textbf {maximize }}&\varvec{ \mu }^\textsf{T} \textbf{w} - \lambda \cdot \textbf{w}^\textsf{T} \Sigma \textbf{w} \\ {\textbf { s.t. }}&\mathbb {1}^\textsf{T} \textbf{w} = 1 \\&\textbf{w} \ge \textbf{0} \\ \end{aligned}, \end{aligned}$$

where

- $\lambda \in \mathbb {R}^+$ is the risk aversion coefficient, i.e., the larger the coefficient, the more risky the generated portfolio, and
- $\textbf{w} \ge \textbf{0}$ defines as positive (short-selling operations are not permitted) value each weight $w_i$.

Hereafter, we will make the following assumptions:

- The total amount available for stock investments is allocated.
- The amount allocated to each stock is kept fixed until the end of the holding time.

The traditional MV model formulation treats the set of input stocks $S$ as a unique, large portfolio and assigns a continuous weight $w_i$ to each stock $s_i \in S$. Conversely, in the present work, we address the binary stock selection problem [29] and rely on a uniform investment strategy. This entails a selection of an equally weighted portfolio over the power set $\mathbb {P}$ of $S$. Moreover, we apply a buy-and-hold strategy to invest in the stock markets (i.e., buy the securities and sell them at the end of the holding time).

## The proposed mean–variance model adaptation

We will present here the adapted version of the traditional MV philosophy, whose goal is not to generate the desired portfolio by shortlisting single stocks, but rather to identify the best portfolio from a set of portfolio candidates.

As a preliminary step, portfolio candidates are generated by means of an itemset-based heuristic presented later on in Sect. 5.4. The idea behind it is to approximate the expected return of a candidate portfolio as a combination of daily returns of the least performing stock in the portfolio. However, notice that the EPP approach can be conveniently generalized and adapted to an arbitrary portfolio-level heuristic that can be computed in a scalable way.

In the itemset-based heuristic, each candidate stock portfolio satisfies a lower-bound estimate of the portfolio return [14] (LBPR, in short), which is defined and computed as follows

4 $$\begin{aligned} \begin{aligned} E_{\min }[ P _q ]= & {} \textsf{average}_{d \in H }\left\{ \textsf{minret}( P _q, d) \right\} , \end{aligned} \end{aligned}$$

where $P_q$ is a selected portfolio identified as an element of the power set $\mathbb {P}$ of $S$, thus $q \in \{1, \ldots , |\mathbb {P}|\}$. The function minret($\cdot$) returns the least daily return over all the portfolio stocks on a given day *d*.

Then, we look for the portfolio $P \in \mathbb {P}$ that is best placed with regard to a single rank-based objective function, where the portfolio evaluation relies on an additional combination of expert-driven decision criteria. Thus, the key idea is to combine the portfolio-level return provided by an ad hoc measure of performance (in this work LBPR) with additional measures of performance of the candidate portfolios that can be independently generated using different strategies (e.g., volatility, wisdom of crowds).

The suggested selection model explores a subset of the portfolios $\mathbb {P}$ and stores into vector^1^$x_q \in \mathbb {P}$ the binary choice relative to each candidate portfolio

5 $$\begin{aligned} \begin{aligned} {\textbf {minimize }}&(1 - \lambda ) \mu ^\mathbb {P} (x_q) + \lambda \cdot \Sigma ^\mathbb {P}(x_q) \\ {\textbf { s.t. }}&\text {c}^\mathbb {P}(x_q) \ge \textsf{Th} \quad \textsf{Th} \in \mathbb {R}^T \\&x_q \in \mathbb {P} \\ \end{aligned}, \end{aligned}$$

where

- The return ranking function $\mu ^\mathbb {P}$: $\mathbb {P} \rightarrow \mathbb {N}^+$ directly interfaces with the adopted portfolio-level heuristic by returning the rank of a candidate portfolio, and in this work, it boils down to a ranking based on the lower bound estimate of the return $E_{\min }[ P _q ]$.
- The risk ranking function $\Sigma ^\mathbb {P}$: $\mathbb {P} \rightarrow \mathbb {N}^+$ returns the rank of a candidate portfolio based on its risk measure, avoiding the estimation of statistical measures for all the combination of available stocks.
- Constraints $\text {c}^\mathbb {P}$: $\mathbb {P} \rightarrow \mathbb {R}^\text {T}$ allow end-users to set up multiple decision criteria based on a variety of *T* factors through a threshold $\textsf{Th}$, among which the stock diversification over sectors, the observed trends in the historical stock prices, and the fundamentals behind the considered assets. Constraint enforcement will be discussed later on (see Sect. 5.3).
- $\lambda \in [0,1]$ is the *risk aversion* of the end-users, which allows us to make a combination of portfolio payoff and risk.

The ranking strategy replaces a combinatorial optimization approach, supplying a meaningful way to compare the generalizations of the risk and the return that could be a priori not comparable with a fully quantitative approach. Notice that in Eq. (5) the dependency on the portfolio family $\mathbb {P}$ is made explicit and the set of additional constraints can be conveniently adapted to the end-users’ needs.

## The early portfolio pruning method

The proposed hybrid method consists of a two-step process:

1. *Candidate portfolio generation* It analyzes historical stock-related data by means of a parallel itemset mining approach to generate a selection of candidate stock portfolios. The aim is to early identify a subset of promising stock portfolios based on a global trend analysis of the composing stock prices.
2. *Portfolio selection* It identifies, among the candidate portfolios generated at the previous step, the best choice according to both a set of end-users preferences and the analysis of additional stock-related data (e.g., fundamental analysis). This step is accomplished by a solver that addresses the adapted mean–variance philosophy described in Sect. 4.

### Data model

We consider the following stock-related data:

- The daily Open-High-Low-Close-Volume (OHLCV) values, associated with each considered stock in the reference time period.
- A taxonomy that clusters stocks into homogeneous categories/financial sectors.
- The financial statements that periodically report the updates of the key economic stock indicators.^2^

OHLCV data are widely used to analyze stock price and volume trends by means of technical analyses as they are expected to inherently incorporate all the underlying effects. They are analyzed in the first step of the hybrid method (candidate portfolio generation).

The taxonomy consists of a set of aggregation hierarchies built over stocks. It is instrumental for diversifying the fund allocation across different sectors thus reducing the overall risk exposure [30].

Financial reports are commonly exploited in fundamental analysis to measure the equity intrinsic value by examining related economic and financial factors [31]. Aggregation hierarchies and financial reports are both used to drive the portfolio selection step.

#### Transactional stock price model

To generate the candidate itemset-based portfolios, the selected heuristic extracts from the OHLCV data the daily closing prices of each of the considered stocks and stores them into a transactional dataset [32]. Each transaction $tr_x$ corresponds to a distinct trading day $d_x$ in the reference time period [$d_{start}$, $d_{end}$]. $tr_x$ consists of the set of pairs $\langle$ $s_i$, $r^x_i$ $\rangle$, where $r^x_i$ is the percentage variation of the closing prices of stock $s_i$ between days $d_x$ and $d_{x-1}$.

An example of transactional dataset is reported in Table 2. It consists of six transactions, each one collecting the closing price variations (w.r.t. the preceding day) of the stocks A, B, C, D, and E on different trading days. For instance, on day $d_1$ the closing price of stock A has increased by 5% w.r.t. to the preceding day. Notice that in transactional data model the temporal order of the contained transactions is not relevant, i.e., the temporal order of day $d_1$-$d_6$ does not matter.

**Table 2:** Transactional data representation. Reference time period [$d_1$, $d_6$]

Time stamp	Transaction
$d_1$	$\langle$A,5%$\rangle$, $\langle$B,5%$\rangle$, $\langle$C,−1%$\rangle$, $\langle$D,7%$\rangle$, $\langle$E,5%$\rangle$
$d_2$	$\langle$A,2%$\rangle$, $\langle$B,6%$\rangle$, $\langle$C,0%$\rangle$, $\langle$D,2%$\rangle$, $\langle$E,2%$\rangle$
$d_3$	$\langle$A,4%$\rangle$, $\langle$B,5%$\rangle$, $\langle$C,−2%$\rangle$, $\langle$D,4%$\rangle$, $\langle$E,5%$\rangle$
$d_4$	$\langle$A,4%$\rangle$, $\langle$B,2.5%$\rangle$, $\langle$C,−4%$\rangle$, $\langle$D,10%$\rangle$, $\langle$E,4%$\rangle$
$d_5$	$\langle$A,1%$\rangle$, $\langle$B,4%$\rangle$, $\langle$C,−2%$\rangle$, $\langle$D,7%$\rangle$, $\langle$E,1%$\rangle$
$d_6$	$\langle$A,−1%$\rangle$, $\langle$B,6%$\rangle$, $\langle$C,0%$\rangle$, $\langle$D,1%$\rangle$, $\langle$E,−1%$\rangle$

#### Taxonomy over stocks

We build a taxonomy over stocks to incorporate the information about stock membership into specific financial sectors. Each stock is mapped to the corresponding sector. In our experiments, the hierarchical stock relationships are derived from the standard GICS sector-based stock categorization.^3^ Alternatively, end-users could automatically infer the relationships using ad hoc clustering-based methods, e.g., [33–35], subspace factorization or genetic algorithms, e.g., [36, 37].

#### Financial statements

Fundamental analysis focuses on examining the economic and financial factors related to a stock (e.g., production, earnings, employment, housing, manufacturing, management). In the current work, we focus on the subset of fundamental factors selected by [38] to forecast stock performance, namely (i) rate of sales growth over the past year (SGI) [39], (ii) gross margin (GMG) [40], (iii) earning surprise (CHGEPS) [41], (iv) total capital expenditures (CAPX), (iv) revenues earned or expenses incurred (ACCRUAL) [42], and (v) level of research and development investments (R &D). However, it is possible to use as constraint other factors as well.

### Candidate portfolio generation

Frequent itemset mining is an established unsupervised technique to discover recurrent item correlations from transactional data [43]. A frequent itemset is an arbitrary set of *l* items ($l \ge$1) whose observed frequency of occurrence (support) is above a given threshold. In our context, itemsets represent arbitrary stock portfolios consisting of *l* stocks.

Traditional itemset mining algorithms such as Apriori [44] and FP-Growth [45] do not consider the weights associated with the items occurring in each transaction. In our context, item weights indicate the percentage closing price variation w.r.t. the preceding trading day.

More recently, various algorithm extensions have been proposed to incorporate item weights into the itemset mining process, e.g., [46, 47]. In parallel, lots of efforts have been devoted to parallelizing the extraction of frequent itemsets using Hadoop–Spark framework in order to scale toward Big datasets, e.g., [13].

The candidate portfolio generator in EPP extracts all the itemsets representing promising stock portfolios by adopting the portfolio-level heuristic evaluator previously proposed by [14]. The key idea is to filter out the combinations whose average least return of the composing stocks is below a given threshold. Since the current implementation of the presented heuristic method is centralized, it is unsuitable in its current form for coping with a very large initial stock set (see Sect. 6.4).

To overcome the above issue, we leverage the parallel and distributed itemset mining techniques presented by [48] and currently supported by the ML-Lib library [49]. Specifically, we tailor the parallel mining process to successfully cope with transactional data including item weights.

### Portfolio selection

Modern financial decision support systems allow end-users to specify their preferences for portfolio selection by different levels of insight, thus extending the original Markowitz’s work that was based only on the first two moments of the distribution of the returns. Decisions are commonly driven by (i) the current market conditions, (ii) the economic investors’ preferences and attitude to risk, and (iii) the intrinsic economic value of the considered assets.

To identify the portfolio, EPP relies on the adapted mean–variance model previously described in Sect. 4. It allows the enforcement of a set of user-specified constraints both at the portfolio-level constraints. Specifically,

- *Fundamental analysis* Portfolios are evaluated in terms of the relative strength of the financial stock fundamentals. The ranking strategy shortlists the portfolios including top-ranked stocks across a variety of established financial indicators.
- *Diversification* Portfolios are expected to include stocks well diversified across sectors and markets. The portfolios that do not meet a sufficient level of diversification are early pruned.
- *Trend* Stock price trends are commonly used to plan trading strategies. Portfolios are shortlisted based on the underlying long-term price trends of the composing stocks, which are estimated using established technical analysis indicators.

A more detailed description of the supported constraints is given below.

#### Portfolio-level constraints based on fundamental factors

We assign a fundamental score to each portfolio based on the characteristics of the composing stocks. Specifically, according to [50], we first derive the financial/economic strength of each stock based on a variety of fundamental factors and then combine the per-stock scores to assign the portfolio-level score.

Starting from the initial set of fundamental indicators/ratios available in the fundamental reports (see Sect. 5.1), we extract a summary consisting of a sample of key financial information. The sample is extracted according to [38]. These values are discussed to be effective on stocks with extreme returns, and thus, they well fit an early pruned family of stocks that have been selected in the first stage of the algorithm by returns. To meet the end-user preferences, the selection process of the considered indicators/ratios is expert-driven. She decides from the default pool the indicators that are worth including.

The per-stock score is an integer number, ranging from zero to the number of activated indicators/ratios. It considers the number of factors that are placed in the upper percentile in the overall stock ranking.^4^ The idea behind it is to appreciate the relative strength of the financial fundamentals of the stock only in terms of relative global quality, by looking at the distribution of each indicator amongst the pruned stocks.

#### Portfolio-level constraints based on diversification

To assess the level of risk of a portfolio, we verify that the selected candidates satisfy a minimum (user-provided) level of stock diversification according to the stock categorization specified in the input taxonomy. Specifically, we compute the diversification level of the portfolio as the percentage of stocks belonging to different categories. Notice that to manage risk exposure the minimum diversification level can be manually specified by the domain expert.

#### Portfolio-level constraints based on technical analyses

Stocks prices can be aggregated and analyzed using the classical technical analysis indicators and oscillators [51] such as Simple Moving Average (SMA) and Exponential Moving Average (EMA). They provide useful information about the underlying stock price trends and exchanged volumes.

To prevent the selection of portfolios that include stocks characterized by negative trends we also incorporate a portfolio evaluation based on technical analysis.

For example, EPP supports the comparison between the current portfolio prices and the simple/exponential moving average at a fixed periodicity (e.g., when the price is above the SMA with period 50 then a price uptrend is likely).

#### Risk aversion

To meet end-users’ preferences, we ask them to set the value of risk aversion $\lambda \in [0,1]$ in the adapted mean–variance logic. The higher $\lambda$, the higher the risk aversion (see Sect. 4).

Notice that alternative, more sophisticated approaches to attach risk aversion levels to stock portfolios (e.g., [52]) can be integrated as well.

### The decision support system

Algorithm 1 presents the proposed decision support system. First, it runs the LBPR algorithm on the dataset of daily stock prices $\mathbf {D_s}$ Then, it ranks the result set shortlisting the top-$\textbf{k}$ portfolios by filtering out all the portfolios with diversification level lower than the given threshold $\mathbf {th_d}$ and with fundamental score below $\mathbf {th_f}$. Finally, it selects the stock portfolio achieving the optimal balance between expected return and risk.

To better clarify the key steps adopted by the decision support system, Fig. 1 shows a sketch of the decision-making process. 
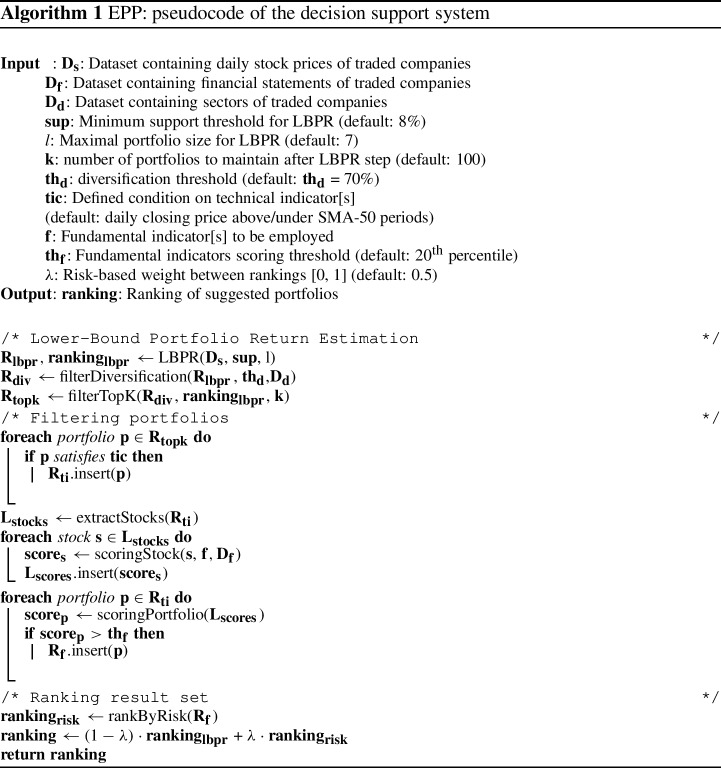

#### Computational complexity

The computational complexity of the LBPR algorithm is mainly influenced by the itemset mining step, which is used to heuristically generate the candidate stock portfolios. The further steps, applied on top of a restricted subset of portfolios, have negligible impact on time and memory complexity.

Enumerating all the possible frequent itemsets in a large dataset is known to be NP-hard [53]. In particular, the number of generated candidates is linear with the dataset size and combinatorial with the number of input items. However, as discussed in [14], the optimal portfolio size is at least one order of magnitude lower than the number of candidate stocks. Hence, its impact is much less critical for maximal itemset mining.

LBPR adopts a parallel implementation of a maximal itemset mining algorithm [13], which guarantees a time complexity of O($\frac{|D_s|}{P}$), where $|D_s|$ is the dataset size and *P* is the number of partitions used in the parallel and distributed computation. Empirical evidence of the algorithm scalability is given in Sect. 6.4.

## Experiments

### Experimental design

#### Data sources

We crawled stock-related data from Yahoo! Finance.^5^

#### Hardware and code

We run the experiments on a hexa-core 2.67 GHz Intel Xeon with 32GB of RAM, running Ubuntu Linux 18.04.4 LTS. The framework is written in the Python and Spark languages. The source code is available for research purposes upon request to the authors.

#### Backtesting

**Fig. 2:**
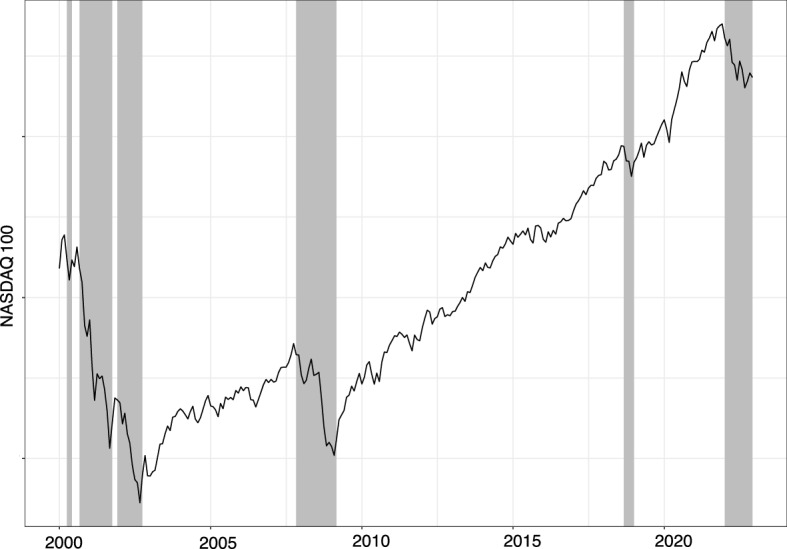
The NASDAQ-100 index. Bearish periods are colored in gray whereas bullish ones are in white

We run a set of backtesting trading simulations to evaluate the profitability and riskiness of EPP. The test periods are defined according to the bearish and bullish market states previously introduced in [54]. To this end, we first segment the raw price series of the analyzed market index into bearish and bullish market states, highlighted in Fig. 2, and then select reference time periods accordingly.

- *Bearish period* Period 2008–2009. It was mainly characterized by a bearish market condition due to the global financial crisis originated by the subprime mortgage crisis.
- *Bullish period* Period 2012–2015. It was characterized by a bullish market condition due to global economic growth. This period is a subsection of the 10-year long bullish period identified. Specifically, the selected subsection is characterized by the fastest growing market of the full period.
- *COVID-19 pandemic period* Period 2018–2020. It was characterized by a mix of bearish and bullish market states. This particular case study is related to the outbreak of the COVID-19 pandemic, the imposition of restrictions, and the end of the first epidemic wave. We consider it as real-life, challenging scenario.

Separately for each period, we run several backtesting simulations to assess the effectiveness and robustness of the portfolio optimization strategies on historical stock-related data relative to the NASDAQ-100 index. Specifically, we learn the itemset-based model using a six-month period (e.g., from July 1, 2007, to December 31, 2007, for the bearish period) and apply it to the next 12 months (e.g., for year 2018). For each simulation, we apply a buy-and-hold strategy, i.e., we buy the portfolio stocks at the beginning of the period and sell them at the end.

In all the performed simulations we consider an initial equity of 100,000 USD, we adopt a fixed-fractional money management strategy (with neither stop loss nor stop profit limits) and approximate per-trade transaction costs to 0.15% [55].

#### Competitors

We compare the performance of EPP with that of

- The **NASDAQ-100 benchmark**, which replicates the NASDAQ-100 index with no leverage.
- The established mean–variance model by Markowitz [55], where, to choose the optimal portfolio on the efficient frontier, we follow the strategy presented by [56] and optimize the choice according to the value of the Sharpe ratio [57], which measures the reward-to-variability ratio of a portfolio compared to a risk-free asset. This portfolio will be identified as **Markowitz–Sharpe** from now on.
- A set of recently proposed deep reinforcement learning (DRL) strategies to stock portfolio allocation available in the **FinRL** library, namely **A2C, TD3, and DDPG** [58].
- The most recently proposed itemset-based heuristic for portfolio generation, namely **DISPLAN** [14].
- Three established **US hedge funds** (only for the most recent COVID-19 pandemic period 2018-2020) investing on the same assets, i.e., MSEGX-Morgan Stanley Inst Growth A,^6^ OLGAX-JPMorgan Large Cap Growth A,^7^ PIODX-Pioneer Fund Class A.^8^

For Markowitz, we generate portfolios by using the *estimateMaxSharpeRatio* function from MATLAB (R2020b). For DISPLAN and EPP, we vary the minimum support threshold in the range [3%,12%], whereas the diversification threshold is set to 70%. To train FinRL, we consider 10 years of historical data to avoid the negative effects of data overfitting.

#### Evaluation metrics

For each trading simulation we analyze

- The **equity line plot**, which graphically shows the temporal variation of the equity during the test period [51].
- The **payout**, which is computed as the overall percentage return/loss of the portfolio at the end of the test period [4].
- The **Volatility**, which measures the standard deviation of the overall portfolio value with regard to the daily returns [4].

We graphically analyze the metrics above by plotting time series representing the percentage variation of the equity w.r.t. the initial value of the investment (e.g., see the equity line in Fig. 6a) and the daily percentage change in the price of the portfolio (e.g., see the volatility plot in Fig. 6b).

#### Scalability

We test the scalability of EPP with both the number of considered stocks and the size of the historical time window used in the learning phase to generate the itemset-based model.

To test the scalability with the number of stocks, we randomly add stocks of the Standard &Poor500 index to the initial stock set and rerun the simulations until the whole S &P500 index is covered.

### Results of the backtesting simulations

**Fig. 3:**
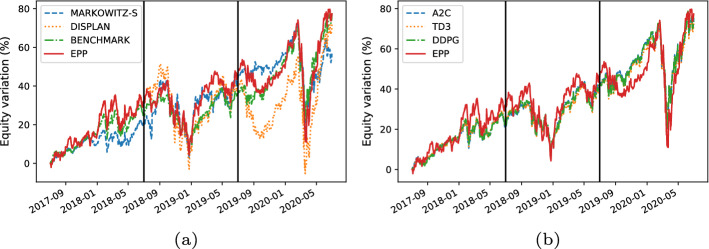
Percentage variation of the equities. COVID-19 pandemic period. NASDAQ-100 index. **a** Comparison with benchmark, DISPLAN, and Markowitz–Sharpe. **b** Comparison with the Deep Reinforcement Learning strategies

**Fig. 4:**
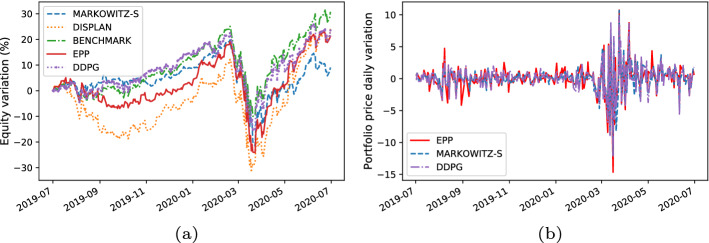
Performance comparison during the outbreak of the COVID-19 pandemic. **a** Percentage variation of the equities. **b** Volatility plot

**Fig. 5:**
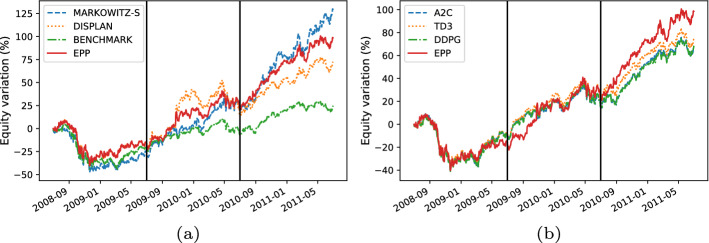
Percentage variation of the equities. Bearish period. NASDAQ-100 index. **a** Comparison with benchmark, DISPLAN, and Markowitz–Sharpe. **b** Comparison with deep reinforcement learning strategies

**Fig. 6:**
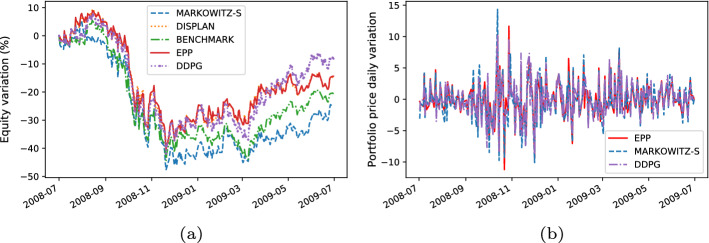
Performance comparison during the outbreak of the financial crisis. **a** Percentage variation of the equities. **b** Volatility plot

**Fig. 7:**
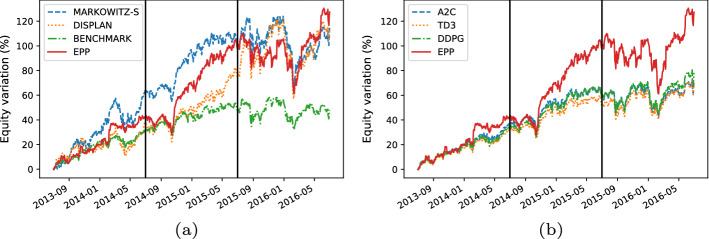
Percentage variation of the equities. Bullish period. NASDAQ-100 index. **a** Comparison with benchmark, DISPLAN, and Markowitz–Sharpe. **b** Comparison with deep reinforcement learning strategies

**Fig. 8:**
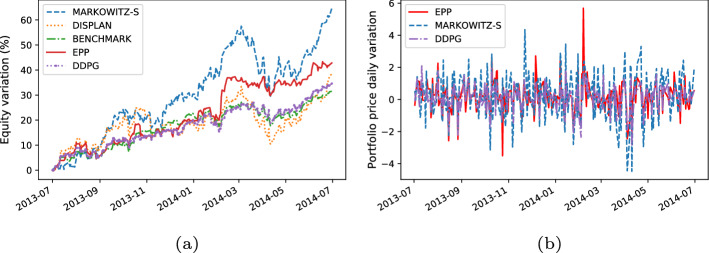
Performance comparison during the period of most significant market growth (2013–2014). **a** Percentage variation of the equities. **b** Volatility plot

**Fig. 9:**
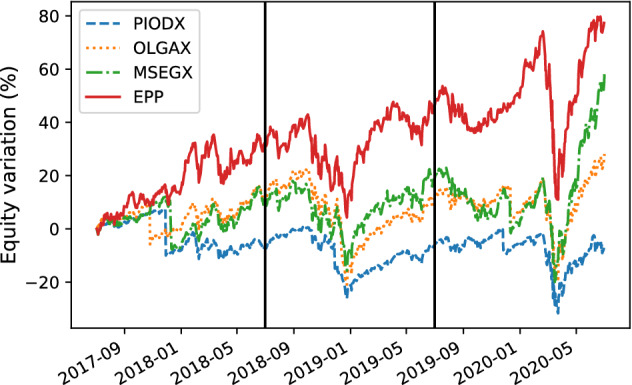
Percentage variation of the equities. COVID-19 pandemic period. Comparison with real funds

Here, we present the following results:

- The comparison between the equity lines of the portfolios generated by EPP and those of the tested competitors (see Figs. 3a, 4a, 5a, 6a, 7a, and 8a). They provide a high-level view of the overall performance achieved by different approaches. For the sake of clarity, the comparisons with the Reinforcement Learning strategies are reported in separate plots (see Figs. 3b, 5b, and 7b).
- The comparison between the equities selected by EPP with those selected by the real hedge funds (see Fig. 9). The aim is to show the applicability of the proposed system in a real scenario.
- The volatility of EPP compared with those of the other approaches (see Figs. 4b, 6b, and 8b).

Hereafter, we will separately analyze each market period.

#### COVID-19 pandemic period

Figure 3a and b compares the equities in the COVID-19 pandemic period (years 2018–2020). EPP shows good resilience properties against negative market trends. For example, note the frequency with which DISPLAN values (dotted in orange) are subject to declines during different temporal windows (e.g., 09/2019-12/2019, 01/2020-05/2020), while EPP maintains profitable values. It outperforms both DISPLAN and Markowitz–Sharpe, while maintaining comparable results with regard to the DRL-based methods. By deepening the analysis of the COVID-19 pandemic outbreak period (see the equity lines in Fig. 4a and the volatility plot in Fig. 4b), EPP and DRL-based methods show a good capability to counteract the market drawdown than DISPLAN and Markowitz–Sharpe during the peak of the epidemic wave. The portfolio capability to be adaptive against adverse market movements is inherent in DRL agents, whereas turns out to be an empirical property of the combination of a static itemset-based model with the adapted Markowitz model.

#### Bearish market period

The analyzed bearish period (2008–2011) is relative to the 2008 financial crisis and the subsequent market recovery. In the aforesaid challenging scenario, EPP maintains a good performance in the first two years (2008–2010), only to be overtaken by Markowitz–Sharpe during the rally following the financial crisis. An opposite result occurs in the comparison with DRL-based models, where many crossovers occur in the first two years, culminating in a final overtaking in the rally phase by EPP. (see Fig. 5a and b). However, by focusing on the outbreak of the financial crisis, the EPP portfolio has shown to be less volatile than Markowitz–Sharpe and its drawdown and payout are roughly comparable to those of the DRL-based methods (see Fig. 6a and b).

#### Bullish market period

EPP performs better than the other tested competitors in the bullish period (see Fig. 7a and b). Deepening the analysis on the period of maximal market growth (see Fig. 8a), Markowitz–Sharpe payout is superior to those of EPP. However, the volatility is significantly higher (see Fig. 8b). The reason is that thanks to the portfolio-level constraints EPP is more conservative than Markowitz–Sharpe even in bullish market conditions when risky strategies that rely on very few stocks are rewarded.

#### Comparison with hedge funds

This confirms the usability of the proposed system in real-world scenarios.

### Effect of the risk aversion

End-users can personalize the risk exposure of the EPP portfolio by conveniently setting the risk aversion $\lambda$. The higher $\lambda$, the more important is the risk-based ranking classification of the candidate portfolios (see Sect. 4).

**Fig. 10:**
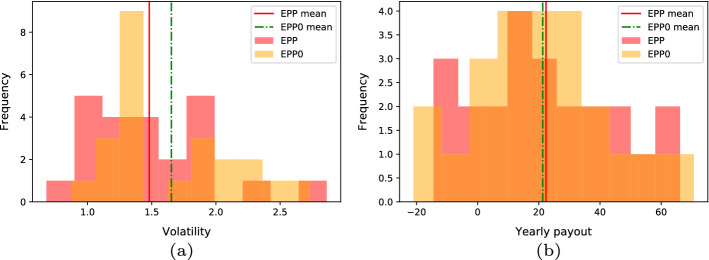
EPP standard configuration with medium risk aversion ($\lambda$=0.5) vs. EPP with no risk aversion ($\lambda$=0). Years 2008-2020. **a** Distributions of the daily volatility statistic. **b** Distributions of the yearly payouts

We run a set of experiments to analyze the effect of the risk aversion on the performance of the generated portfolios. Figure 10a and b compares the daily volatility and payout distributions over all the analyzed years (2008–2020) achieved by setting a medium risk aversion ($\lambda =0.5$) and no risk aversion ($\lambda =0$), respectively. The mean payout values are roughly comparable with each other, whereas the volatility of the configuration setting with no risk aversion is consistently higher. However, setting an extreme configuration is not advisable because, on the one hand, taking into account no risk rankings may expose investors to more relevant market oscillations without yielding significant returns. On the other hand, the risk ranking alone would erase the pivotal role of the selected heuristic.

### Scalability tests

We run several backtesting simulations using the EPP method to test the scalability with the number of considered stocks and the size of the training window.

Firstly, we tested EPP on a larger set of stocks, i.e., the entire S &P 500 index (500 stocks). Figure 12 shows the equities lines of both EPP and the benchmark S &P 500 index over the COVID-19 pandemic period. The results confirm the effectiveness of the proposed strategy.

**Fig. 11:**
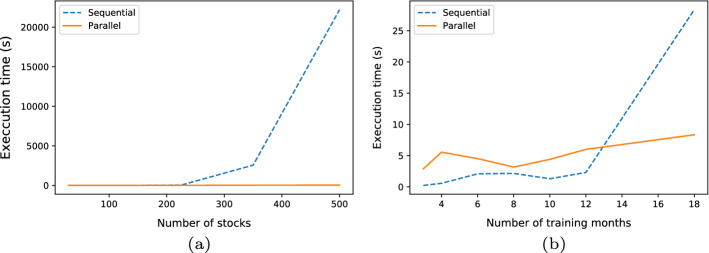
Time complexity analysis. Comparison between EEP with parallel itemset mining with the EPP variant with sequential itemset mining. **a** Scalability with the number of initial stocks. **b** Scalability with the training window size

Secondly, we vary the number of initial stocks from 10 to 500 to test the system scalability (see Fig. 11a). To evaluate the impact of the parallel itemset mining phase on the EPP time complexity, we test both a parallel and a centralized version of the system. In the centralized variant of EPP, we replace the PFP algorithm [13] with an efficient, centralized FP-Growth algorithm implementation.^9^

As expected, the increase in the number of initial stocks results in a super-linear increase of the execution time, mainly due to the generation of a combinatorial number of candidate itemsets, which are then processed in a sequential manner. Conversely, in the parallel version the job is distributed across multiple workers and the increase is approximately linear with the number of stocks.

Figure 11a shows a similar scalability test executed by keeping the number of initial stocks fixed to 100 (i.e., the stocks in the NASDAQ-100 index) and by varying the number of training months from 3 to 18. A nonlinear increase in the time complexity comes out by considering a training window size larger than 12. However, the latter scalability issue seems to be less critical than the former one as the standard window size configuration is typically between 6 and 12 months.

**Fig. 12:**
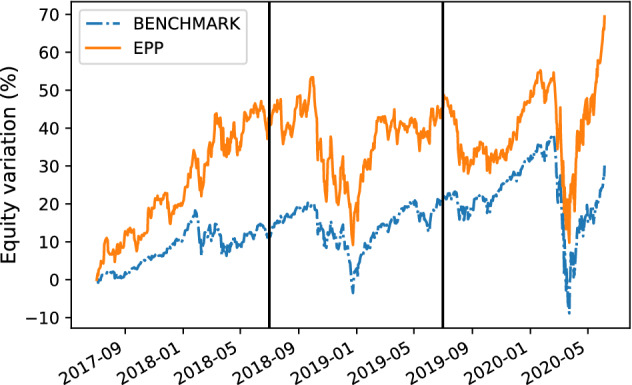
Percentage variation of the equities. COVID-19 pandemic period. S &P 500 stocks. Comparison between EPP and the benchmark

## Conclusions and future works

The paper presented a hybrid financial decision support system for selecting stock portfolios. The framework allows for the combination of a parallel itemset mining process applied to historical stock price data with a tailored set of constraint and a risk-averse adjustment. The key idea is to simplify the complexity of stock-based approaches by early filtering part of the candidate portfolios during the initial itemset mining phase. Specifically, the extracted itemsets represent candidate stock portfolios, where we directly apply the traditional Markowitz’s philosophy, allowing also for the enforcement of further portfolio-level constraints based on complementary knowledge provided by taxonomies and financial reports.

The integration of a parallel itemset mining allows for the analysis of large stock sets not manageable by a centralized approach and the selected portfolios achieves good performances in terms of payout and risk exposure when compared to:

- Deep reinforcement learning methods. (Even if EPP relies on static stock portfolios, whereas DRL dynamically adapts the model to the current market situation.)
- Markowitz–Sharpe models.
- Established US hedge funds.

As future work, we plan to: (i) Extend the current hybrid method and DSS by integrating financial instruments other than stocks (e.g., exchange-traded funds); (ii) test the proposed approach on non-US stocks by simulating multinational scenarios and properly managing both multinational financial data and the geographical diversification over stocks; (iii) improve the way decision-makers reflect their risk aversion; and (iv) investigate the use of fuzzy rule models [59] and probabilistic itemset mining [60] to model market uncertainty.

## Data Availability

The datasets generated during and/or analyzed during the current study are available from the corresponding author upon request.

## Footnotes

1 Notice that for each portfolio $P_q$ holds a biunivocal relation with the binary vector $x_q$ with 1 when the stock $s_i$ is selected in portfolio $P_q$ and 0 otherwise.

2 In the experiments, we will consider the quarterly reports published by **Yahoo! Finance** and available at https://finance.yahoo.com/ (latest access: December 2021).

3 https://www.msci.com/gics (latest access: December 2021).

4 In compliance with [50] in the experiments we rank the stocks by decreasing summarizing factor score and then pick only those in the upper 80% percentile.

5 https://finance.yahoo.com. (Latest access: August 2020).

6 https://www.morganstanley.com/im/en-us/intermediary-manager-research/product-and-performance/mutual-funds/us-equity/growth-portfolio.shareClass.A.html (Latest access: March 2022).

7 https://am.jpmorgan.com/us/en/asset-management/adv/products/jpmorgan-large-cap-growth-fund-a-4812c0506 (Latest access: March 2022).

8 https://www.amundi.com/usinvestors/Products/Mutual-Funds (Latest access: March 2022).

9 http://fimi.uantwerpen.be/src/ (Latest access: December 2021).
